# Surveillance for highly pathogenic H5 avian influenza virus in synanthropic wildlife associated with poultry farms during an acute outbreak

**DOI:** 10.1038/srep36237

**Published:** 2016-11-04

**Authors:** Susan A. Shriner, J. Jeffrey Root, Mark W. Lutman, Jason M. Kloft, Kaci K. VanDalen, Heather J. Sullivan, Timothy S. White, Michael P. Milleson, Jerry L. Hairston, Shannon C. Chandler, Paul C. Wolf, Clinton T. Turnage, Brian J. McCluskey, Amy L. Vincent, Mia K. Torchetti, Thomas Gidlewski, Thomas J. DeLiberto

**Affiliations:** 1 National Wildlife Research Center, USDA-APHIS, Fort Collins, CO, USA; 2 Wildlife Services, USDA-APHIS, Manhattan, KS USA; 3 Wildlife Services, USDA-APHIS, Springfield, IL USA; 4 Wildlife Services, USDA-APHIS, Gainesville, FL USA; 5 Wildlife Services, USDA-APHIS, Raleigh, NC USA; 6 Wildlife Services, USDA-APHIS, Sacramento, CA USA; 7 Wildlife Services, USDA-APHIS, Minneapolis, MN USA; 8 Wildlife Services, USDA-APHIS, Sherwood, AR USA; 9 Science, Technology and Analysis Services, Veterinary Services, USDA-APHIS, Fort Collins, CO, USA; 10 Virus and Prion Diseases of Livestock Research Unit, National Animal Disease Center, USDA-ARS, Ames, Iowa, USA; 11 National Veterinary Services Laboratories, Science, Technology and Analysis Services, Veterinary Services, USDA-APHIS, Ames, IA, USA

## Abstract

In November 2014, a Eurasian strain H5N8 highly pathogenic avian influenza virus was detected in poultry in Canada. Introduced viruses were soon detected in the United States and within six months had spread to 21 states with more than 48 million poultry affected. In an effort to study potential mechanisms of spread of the Eurasian H5 virus, the United States Department of Agriculture coordinated several epidemiologic investigations at poultry farms. As part of those efforts, we sampled synanthropic birds and mammals at five infected and five uninfected poultry farms in northwest Iowa for exposure to avian influenza viruses. Across all farms, we collected 2,627 samples from 648 individual birds and mammals. House mice were the most common mammal species captured while house sparrows, European starlings, rock pigeons, swallows, and American robins were the most commonly captured birds. A single European starling was positive for Eurasian H5 viral RNA and seropositive for antibodies reactive to the Eurasian H5 virus. Two American robins were also seropositive. No mammal species showed evidence of infection. These results indicate synanthropic species merit further scrutiny to better understand potential biosecurity risks. We propose a set of management practices aimed at reducing wildlife incursions.

In November 2014, two poultry farms (chickens and turkeys) in the Fraser Valley of British Columbia, Canada^1^ were the first of hundreds of farms in North America to be confirmed with H5 highly pathogenic avian influenza (HPAI) during a seven-month period. These two Canadian poultry farms were located in Abbotsford, a municipality sharing a border with the United States (US). Given the proximity of the detections, the US Departments of Agriculture and Interior (USDA and USDOI), along with state agencies, initiated heightened surveillance operations^2^. Just under two weeks after the World Organisation for Animal Health (OIE) reported the poultry outbreaks in the Fraser Valley, the US filed an OIE report for nearby Whatcom County, Washington disclosing H5N2 HPAI virus (HPAIV) detected in a wild pintail (*Anas acuta*). Concomitantly, H5N8 HPAIV was detected in a captive gyrfalcon (*Falco rusticolus*) fed hunter harvested waterfowl from the same county^2^. Three days later, H5N8 HPAIV was detected in a small backyard farm with 130 mixed birds in Douglas County, Oregon. The H5N8 viruses were consistent with other 2014 Eurasian H5 clade 2.3.4.4 viruses based upon whole genome sequence; the H5N2 virus from Canada and the US was a Eurasian-American (EU/AM) reassortant (five EU genes including H5 and three AM genes including the N2)^2^. In early 2014, outbreaks of H5 clade 2.3.4.4 (aka intercontinental group A [icA] viruses) were reported in Asia, subsequently Europe, and by late 2014, for the first time in North America^3^.

Surveillance efforts continued to identify the Eurasian H5 icA viruses in wild birds in a number of western states (Washington, Oregon, California, Utah, Idaho)^4^ and less than two months after the first detection in the US, H5N8 HPAIV was detected in a commercial turkey flock in California, followed by a second detection in a commercial chicken flock approximately three weeks later. Just over two weeks after that, and in what was to be the first of more than one hundred affected premises in the state, the reassortant EU/AM H5N2 HPAIV was detected in a commercial turkey farm in Minnesota^1^. By mid-April 2015, more than 25 commercial farms in Minnesota were affected. At that point, the virus was detected in the first large commercial chicken egg-layer flock in Iowa, a farm with well over four million birds. The virus continued to spread among Iowa farms until, finally, in mid-June of 2015, the last detection in a commercial poultry operation was reported. In Iowa, six counties suffered poultry losses in the hundreds of thousands, while another six counties suffered losses in the millions (Fig. 1). All told, greater than 48 million poultry were affected in the US, with approximately 9 million birds dead or culled on Minnesota farms and more than 31 million birds dead or culled on more than 70 commercial Iowa farms. While calculating the full costs of the US outbreaks will take considerable time, initial estimates indicate losses in the billions (US dollars). Early estimates indicate the US federal government has spent nearly one billion dollars^5^,^6^ and an analysis commissioned by the Iowa Farm Bureau estimated the impact on that state to be $1.2 billion^7^.

In an effort to study potential mechanisms of introduction or spread of the Eurasian H5 viruses into commercial operations, the USDA coordinated several epidemiological investigations at poultry farms. Outbreaks of HPAI have been relatively rare in the US; thus, emergency response efforts need to include proactive epidemiological investigations that not only investigate the patterns and determinants of an outbreak, but also gather real-time data that can be used to assess risk and inform management practices to improve biosafety protocols. As part of these efforts, we sampled wild synanthropic birds and mammals associated with egg-layer chicken farms in northwest Iowa for exposure to influenza A viruses (IAVs, Fig. 2). While wild aquatic birds have long been considered the natural maintenance hosts of avian IAV^8^, increasing attention has been focused on synanthropic wildlife as spillover hosts that could act as bridge hosts, potentially capable of moving these viruses from natural maintenance hosts to poultry or between poultry farms. Previous studies of peridomestic mammals^9^,^10^,^11^,^12^,^13^,^14^,^15^,^16^,^17^ and birds^16^,^18^,^19^,^20^,^21^,^22^,^23^,^24^,^25^,^26^,^27^,^28^,^29^,^30^,^31^,^32^ have provided strong evidence that a number of these species are able to shed high levels of some IAVs. Nonetheless, only a few studies^10^,^13^,^31^ have examined the potential of any of these species to transmit influenza A viruses to poultry species. Similarly, field assessments of these species on farms are rare^33^,^34^,^35^.

## Results

### Sampling results

**Figure 2:**
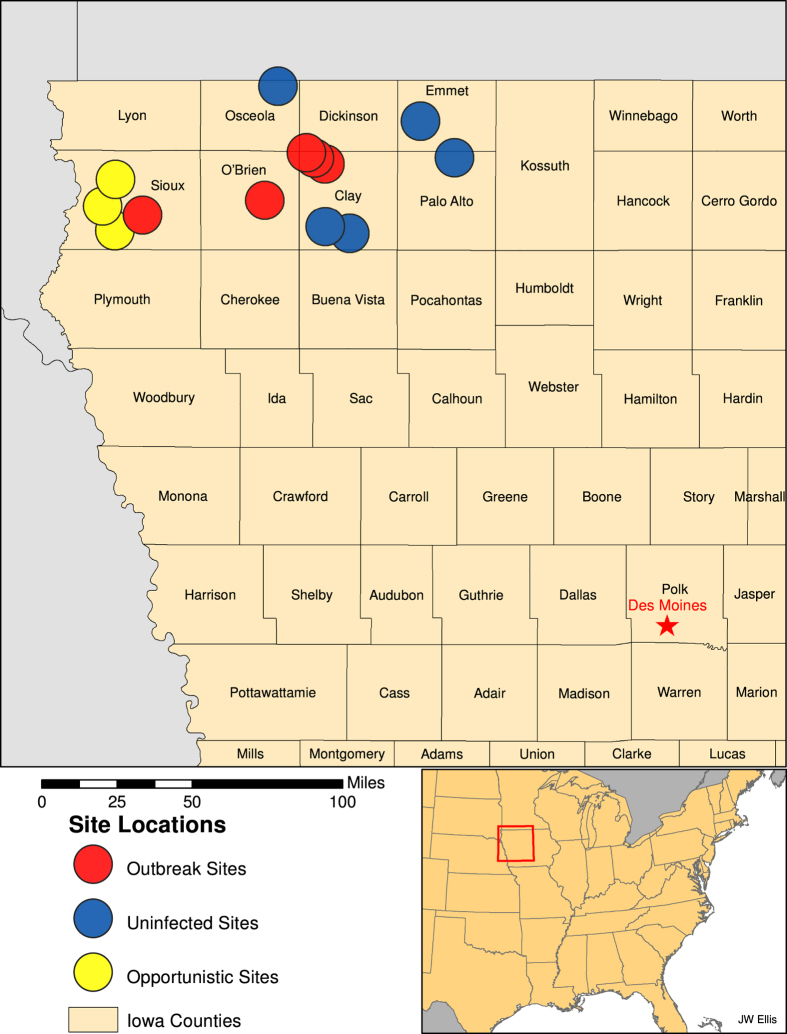
Locations of H5N2 HPAI outbreak and uninfected commercial chicken farms where wild birds and mammals were sampled.

Across ten sampled farms (five infected, five uninfected, Table 1, Fig. 2) and some opportunistic collections at three additional outbreak farms, we collected 2,627 samples from 648 individuals (Table 2). On infected facilities, we collected samples from 190 individual mammals from three species (primarily house mice, *Mus musculus*) and 220 individual birds across 17 species and on uninfected farms, we collected samples from 39 individual mammals from five species (primarily mice) and 199 individual birds across 18 species (Table 3). House sparrows (*Passer domesticus*), European starlings (*Sturnus vulgaris*), rock pigeons (*Columba livia*), American robins (*Turdus migratorius*), common grackles (*Quisculas quiscula*), cliff swallows (*Petrochelidon pyrrhonota*), and barn swallows (*Hirundo rustica*) were the most commonly sampled bird species and accounted for 88% of birds captured (Table 3). The remaining birds sampled (not included in Table 3) were from 16 species (representing 3 orders). These included nine red-winged blackbirds (*Agelaius phoeniceus*), nine chipping sparrows (*Spizella passerine*), six American goldfinches (*Spinus tristis*), six brown-headed cowbirds (*Molothrus ater*), five common yellowthroats (*Geothlypis trichas*), four killdeer (*Charadrius vociferous*), three least flycatchers (*Empidonax minimus*), three vesper sparrows (*Pooecetes gramineus*), two American redstarts (*Setophaga ruticilla*), two gray catbirds (*Dumetella carolinensis*), and one each of Eastern bluebird (*Sialia sialis*), blue jay (*Cyanocitta cristata*), Eastern kingbird (*Tyrannus tyrannus*), ring-necked pheasant (*Phasianus colchicus*), Savannah sparrow (*Passerculus sandwichensis*), and yellow warbler (*Setophaga petechia*).

### Influenza virus detection

Of the 2,184 swab, nasal wash, and tissue samples tested, IAV RNA was detected in a single sample by real-time reverse transcriptase polymerase chain reaction (RRT-PCR) targeting two different genes (the matrix gene and the hemagglutinin gene using Eurasian icA-H5 specific primers). The sample was from lung tissue collected from a juvenile European starling captured on Farm 5. We sampled on Farm 5 approximately four weeks after clinical signs first appeared in poultry. Depopulation was complete prior to our sampling efforts and carcasses were primarily being held in sealed bags in roll-off dumpster containers prior to final disposition. Sample volume was insufficient for virus isolation; an attempt was made to isolate the Eurasian H5 virus using diluted sample, but was not successful.

### Serology results

Serological screening of avian serum samples using IAV ELISA identified seven positive samples and eight suspect positive samples from house sparrows, European starlings, common grackles, American robins, and rock pigeons captured at five premises (Table 4). Of the 15 potential positive samples, three of the serum samples were positive by hemagglutinin inhibition (HI) test for previous exposure to the icA-H5 virus. Two of these samples were from American robins and one was from the same European starling from which icA-H5 specific influenza viral RNA was detected. Two additional serum samples, one from a European starling and one from a house sparrow, were not reactive to the icA-H5 strain, but both of these samples showed low reactivity by the neuraminidase inhibition (NI) test to one or both of a H7N2 virus and a H9N2 virus, suggesting exposure to IAV, possibly an N2. Each of these samples were from Farm 5, as were four additional birds that were positive only by the screening assay: two common grackles and a European starling were positive and an additional European starling was suspect positive. Three samples from rock pigeons collected at two of the opportunistic sites (Fig. 2) and one sample from a house sparrow from Farm 2 were suspect positive by the screening assay. Finally, two samples from European starlings collected from two different uninfected farms were suspect positive for exposure to IAV by the screening assay. Apart from the three HI positive samples that were indicative of exposure to the icA-H5 virus, no subtype confirmation by HI or NI testing was obtained for the other samples positive or suspect positive by the screening assay. All mammal samples were negative for antibody to the icA-H5 virus.

## Discussion

Of 2,185 swab, wash, or tissue samples from 648 individuals, no IAV was recovered from any of the synanthropic wildlife species tested. However, we did identify the presence of viral RNA associated with the highly pathogenic Eurasian origin H5 outbreak virus in a single synanthropic bird associated with a poultry farm in northwest Iowa. This sample was collected from the lung tissue of a juvenile European starling associated with a nest built in a breach of the exterior siding of a raised and enclosed walkway between two poultry buildings. While the nest was located in a breach, there was no indication that the birds associated with the nest were able to access the walkway interior. Nonetheless, this finding suggests further investigation of European starlings is warranted to determine if this species could play a role in the epidemiology of outbreak avian IAVs, as it is a common species found on farms. This same individual starling was also positive for antibodies to IAV by the screening assay and HI test, suggesting exposure to an icA-H5 virus. Because this individual was positive for both icA-H5 viral RNA and seropositive for antibodies reactive to that virus, it is likely that the bird was sampled later in the infection curve when virus recovery is less likely.

In addition to the RNA positive/seropositive starling, two American robins also showed serologic evidence for exposure to the icA-H5 virus; all three of these birds were from the same infected premises. Other serologic evidence for exposure to the outbreak virus in synanthropic birds includes samples from a second European starling and a house sparrow which had low reactivity by NI to N2. In addition, two common grackles, five other starlings, two house sparrows, and three rock pigeons all showed evidence of prior exposure to IAV by the screening assay. These species are commonly associated with farms and livestock operations and have been previously identified as target species that have the potential to act as bridge hosts that move influenza viruses into poultry^27^. Moreover, conclusions from epidemiological questionnaires administered by state and federal officials to outbreak farm managers indicate the need for research characterizing IAV infection dynamics in small perching birds^6^.

Many factors may have contributed to the relatively low number of positive samples found in our surveillance. To begin with, we sampled 648 individuals across 10 farms, five of which were uninfected. On infected farms we sampled 190 mammals and 220 birds which averages out to fewer than 50 mammals or birds per farm. Given that many of the synanthropic species targeted are very abundant on farms, this is a relatively moderate sample size. Another potentially limiting factor may have been our relatively late arrival on farms. Four of the five outbreak farms were depopulated prior to our sampling. If the virus was circulating in synanthropic species prior to the outbreak, most infections would have waned before our sampling. The same is likely true if the virus had moved into these species from poultry. Moreover, because we were sampling in spring, many of the mice and birds that we sampled were juveniles that were likely born or hatched after peak infections. Further elucidation of the role of potential bridge hosts will require sampling on farms as soon as an outbreak is identified. Proactive surveillance of peridomestic species on farms during peak influenza seasons for maintenance hosts would also provide important information on the prevalence of influenza viruses in synanthropic species.

Other factors that may have limited the number of positive samples we identified include reduced capture probabilities if infected individuals become ill and are less likely to be caught, or potential mortality associated with infection which would have removed individuals from the population for sampling. While there are some published studies on the impact of HPAI viruses on synanthropic birds, the impact of the icA-H5 virus strains on these birds is unknown. Experimental infections of laboratory mice with icA-H5 virus strains indicate that infection with these viruses results in very limited morbidity, mortality, and replication compared with highly pathogenic Asian H5N1 viruses^36^. Further, while the serological test used for screening for antibodies to IAV has been shown to be broadly effective for multiple wildlife species^37^, it is possible that the sensitivity of the test may be different from published values for the sampled species.

Disease and outbreak research at the wildlife-livestock interface has increased over time as evidenced by a significant increase in publications on the wild bird-poultry interface starting in 2003. This increase was associated with the rise of H5N1 HPAI in Asia^38^. The first conceptual model of risk pathways describing potential synanthropic wildlife species that could potentially transport IAVs from maintenance hosts (Anseriformes and Charadriiformes) onto farms was published in 2006^13^. More recently, researchers have begun to identify methods for prioritizing target bridge host species for IAV surveillance^27^,^39^. In a comprehensive review of bridge hosts in disease ecology, with a focus on IAVs, bridge hosts were defined as non-maintenance host species capable of transmitting a pathogen from a reservoir population to a target population^40^. By this definition, bridge hosts for IAV come into contact with both maintenance hosts (aquatic birds) and/or their habitat and poultry and/or their environment. Contact between bridge host species and maintenance hosts could occur via shared resources, such as water in streams, drainages, or ponds or via shared use of fields surrounding poultry barns which are used by many waterfowl hosts feeding on corn and other crops.

Bridge hosts have the potential to transmit pathogens directly to poultry by actively shedding virus or by mechanical spread of infectious material^27^. These species could transport viruses by directly entering barns through roof vents, fan vents, breaches in barn buildings, or opportunistically through open doors. Alternatively, they could contaminate feed, water, or the environment and these materials could subsequently be brought into contact with poultry during the movement of poultry workers or associated equipment and supplies. Another potential mechanism leading to an infected barn could be a scenario in which a series of events sparks an outbreak. For example, although we found no evidence that the icA-H5 RNA detected in the lung tissue of the European starling sampled during the current study had access to the barn walkway interior or was actively shedding virus, it is not inconceivable that virus brought to the perimeter of a farm by one species could be subsequently trafficked to the interior of a building by another means.

Mice were commonly observed in some barns during our surveys on infected premises, and were seen moving among cages within poultry buildings as well as on conveyor belts within and between buildings. These observations may have been artificially high on some farms due to limited human activity in the barns post depopulation and because some of the farms reduced active rodent control once depopulation efforts were complete. Rodent control programs are a necessary part of biosecurity programs in poultry farms^41^ and insufficient rodent control has been suggested as one of many potential pathways leading to the establishment of avian influenza viruses on farms^42^. Consequently, ensuring robust rodent control programs may be an important strategy to limit the potential transmission of IAVs by these animals. Surveillance for mice in response to an H5N2 outbreak in Pennsylvania in 1983^35^ and our sampling of mice did not detect any evidence of infection which may indicate that these animals represent a low risk as a bridge host for these strains of IAV. However, our surveillance efforts were conducted weeks to months after the outbreaks which may skew results for a relatively short-lived and highly fecund species. Because mice and other mammals have been identified as a potential risk path for virus transmission within and between farms^9^,^16^,^43^,^44^,^45^,^46^, proactive surveillance and an assessment of multiple viral strains is necessary to rule out the risk these species may pose.

The detection of icA-H5 specific RNA in a synanthropic bird has potential implications for IAV intrusions within a farm and may also have implications for virus trafficking between farms. European starlings have been shown to move between multiple farms (feed lots) rather than exclusively using a single farm^47^,^48^. While rock pigeons have generally been shown to be refractory to IAVs^49^, they are also known to visit multiple livestock farms and have been proposed as potential vehicles of pathogen spread among farms^50^. The potential risk posed by these species likely varies as a function of within-host dynamics (e.g., replication-competence, shedding levels), individual behavior, physiology, season (e.g., breeding or not), and weather (e.g., temperatures^51^).

While a number of authors have studied the prevalence of IAVs in passerines (reviewed in ref. 52), synanthropic birds have not been a primary sampling focus. In those studies, serological or molecular detections have been found in a number of peridomestic species: American robins^32^,^52^, barn swallows^53^, European starlings^18^,^54^,^55^,^56^,^57^. house sparrows^58^,^59^, rock pigeons^25^,^60^, and American crows (*Corvus brachyrhynchos*)^61^. A majority of these detections were associated with anthropogenically modified landscapes. A significant amount of sampling of passerines has taken place in natural settings and in settings where maintenance hosts are resident, but very limited sampling has taken place in habitats frequented by waterfowl that are on or adjacent to poultry farms. Understanding whether synanthropic birds act as bridge hosts requires that sampling takes place both on poultry farms and associated wetlands and water bodies. We have observed that water bodies of various types such as intermittent puddles, swales, and human-made ponds are common on many poultry farms. In some instances, these areas are frequented by both waterfowl and synanthropic wild birds. In addition, mammals also commonly use these water resources (as assessed by observations of tracks and scat).

**Figure 3:**
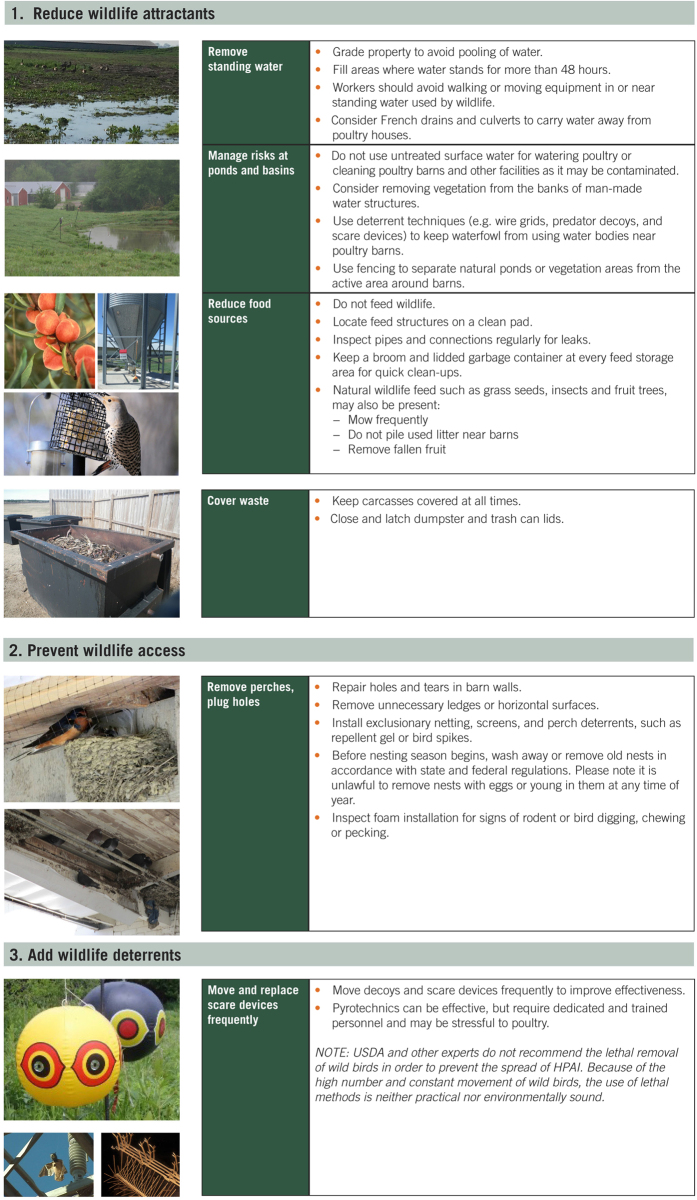
Wildlife management practices that can improve biosecurity measures to help prevent wild birds and other wildlife from coming into direct contact with poultry and to prevent fecal material and secretions from being transported on boots, equipment, and food.

Whereas most large commercial farms have biosecurity measures in place to reduce wildlife incursions, completely eliminating incursions remains elusive and the full suite of biosecurity measures that can be used to prevent IAV incursions from wildlife are not widely known. We developed a set of wildlife management practices on farms aimed at reducing the potential spread of IAVs via synanthropic wildlife. These practices can be divided into three main areas of action: 1) reducing wildlife attractants, 2) preventing wildlife access, and 3) utilizing wildlife deterrents (Fig. 3). These practices center on minimizing contact with maintenance host species such as ducks and shorebirds as well as identifying practices that reduce the probability that a bridge host would move a virus from a maintenance host species to poultry.

Although best management practices of excluding and deterring wildlife from farms are important facets of biosecurity plans for poultry operations, the identification of wildlife species that frequently visit farms is also a high priority^27^. For example, a potential wildlife bridge host that is replication-competent and/or has the capacity to mechanically transport IAVs into farm settings is a low risk candidate in the epidemiology of these viruses on farms if they infrequently visit these facilities. Thus, the identification of key bridge host species is not a trivial task since both field and laboratory investigations are required to truly assess the risk posed by these animals.

While the current study was unable to document that the synanthropic species sampled served as bridge hosts or mechanical vectors of the icA-H5 virus on the farms sampled, we did document the detection of icA-H5 specific RNA in one bird as well as several seropositive birds. Although these numbers are somewhat low given the number of individual animals screened, 40% of the sampled animals were from uninfected farms. Also, some infected farms had been completely depopulated by the time we collected samples while others were at varying stages of depopulation. This temporal lag may have reduced the probability of detection. If future outbreaks occur, real-time wildlife sampling may provide better information on the potential role of synanthropic wildlife in avian IAV epidemiology. Identifying these hosts and the risk they may pose to commercial poultry operations remains an understudied subject in our understanding of IAV outbreaks.

## Methods

### Flocks

We sampled birds and mammals at five chicken layer farms with confirmed EU/AM H5N2 HPAIV and five chicken layer farms with no known infections with HPAIV (Fig. 2, Table 1). All flocks were located in northwest Iowa. Two of the infected farms and two of the uninfected farms were pullet farms (young birds). Sampling at confirmed infected sites was conducted 2–4 weeks after clinical signs were evident in poultry (Table 1). Four of the five infected flocks were primarily depopulated prior to the onset of wildlife sampling and depopulation was concurrent with wildlife sampling at one of the infected farms. Poultry farms with no known infections were generally matched to outbreak farms and exhibited relatively similar flock sizes compared with infected farms (i.e., two moderate, one large, and two very large commercial flocks).

### Sampling Procedures

Wild birds and wild mammals were captured on farms, primarily in and around farm structures. Birds were sampled using mist nets, baited funnel traps, and air guns. Nets and bird traps were opened in the morning, checked frequently and closed in the afternoons. Mammals were trapped using baited collapsible Sherman traps (small rodents) and baited Tomahawk traps (5″ × 5″ × 16″ for cottontails, 10″ × 12″ × 32″ for mesocarnivores). Traps were set in the afternoon and checked in the morning. Some Sherman traps were placed inside poultry houses, but only on infected farms. In addition, several fresh wild bird carcasses found associated with the poultry operations were also sampled. Samples were collected in accordance with the USDA FY2016 HPAI Response Avian Sample Collection for Influenza A and Newcastle Disease, WI-AV-0020. Bird samples were collected in accordance with the guidelines and regulations set forth by the United States Fish and Wildlife Service (USFWS) under permit #MB084762-1, and all samples were collected with permission of the poultry industry.

We collected samples from captured animals to test for IAV and/or antibodies to IAVs. For birds, we collected oral and cloacal swabs. For some individuals, we also collected external swabs by gently wiping a pre-moistened swab on bills, feet, and feathers. For targeted avian species (e.g., house sparrows, European starlings), we collected both a blood sample and lung tissue and for others only a blood sample was collected (e.g., American robins). For mammals, we collected oral and nasal swabs/washes as well as external swabs for some individuals. For targeted species (e.g., mice), we collected a blood sample and lung and/or trachea tissue samples. Further, we collected any observed aberrant tissue (e.g., lesion, abnormal mass). Swabs, nasal washes, and tissue samples were placed in 1–3 mL of transport media (BHI: brain-heart infusion broth) and stored on ice. Blood was collected into serum separator tubes, allowed to clot, and centrifuged prior to shipping. We maintained a cold chain for all samples and shipped samples to testing laboratories within 24 hours during the week or samples were stored in a refrigerator at 4 °C for later shipping for samples collected on Fridays-Sundays. All samples were shipped overnight on ice.

### Laboratory Procedures

Swabs, nasal washes, and tissue samples were screened for IAV matrix gene RNA by RRT-PCR^62^. The Avian Veterinary Diagnostic Laboratory at Colorado State University conducted RRT-PCR testing of avian oral and cloacal swabs, while the National Wildlife Research Center Virology Laboratory conducted RRT-PCR on all other samples. Per the National Animal Health Laboratory Network protocol, any non-negative cycle threshold (Ct) or cycle quantity (Cq) value is submitted for confirmatory testing. Therefore, samples with non-negative Ct/Cq values by matrix gene RRT-PCR were submitted to the USDA National Veterinary Services Laboratory (NVSL) in Ames, Iowa for confirmatory testing. Confirmatory testing included subtype confirmation using routine H5 and H7 specific RRT-PCR assays designed to detect a broad range of Eurasian, Mexican and North American lineage viruses, as well as the icA-H5 specific RRT-PCR assay which targets the Eurasian H5 clade 2.3.4.4 viruses (aka icA) first detected in the US in December 2014. Virus isolation in embryonated chicken eggs was conducted in parallel. Avian serum samples with adequate sample volumes were screened for antibodies to IAV using the IDEXX AI Multi-S Screen Ab test, which is a multi-species blocking enzyme-linked immunosorbent assay (ELISA) targeting an epitope of the IAV nucleoprotein. Because this test has not been specifically validated for the tested species, samples with Sample to Negative (S/N) ratios ≤0.5 were considered positive and samples with S/N ratios between 0.5 and 0.6 were considered suspect positive^63^. All avian and mammalian serum samples with adequate volumes were submitted to NVSL for testing by HI using the icA-H5 virus as the antigen. Avian samples that were antibody-positive by the IDEXX Multi-S test, but negative by HI to the icA-H5 (and where sufficient serum was available), were additionally tested using a standard North American panel of H1-H16 and by NI^64^.

## Additional Information

**How to cite this article**: Shriner, S. A. *et al*. Surveillance for highly pathogenic H5 avian influenza virus in synanthropic wildlife associated with poultry farms during an acute outbreak. *Sci. Rep.* **6**, 36237; doi: 10.1038/srep36237 (2016).

**Publisher’s note:** Springer Nature remains neutral with regard to jurisdictional claims in published maps and institutional affiliations.

## Figures and Tables

**Figure 1:**
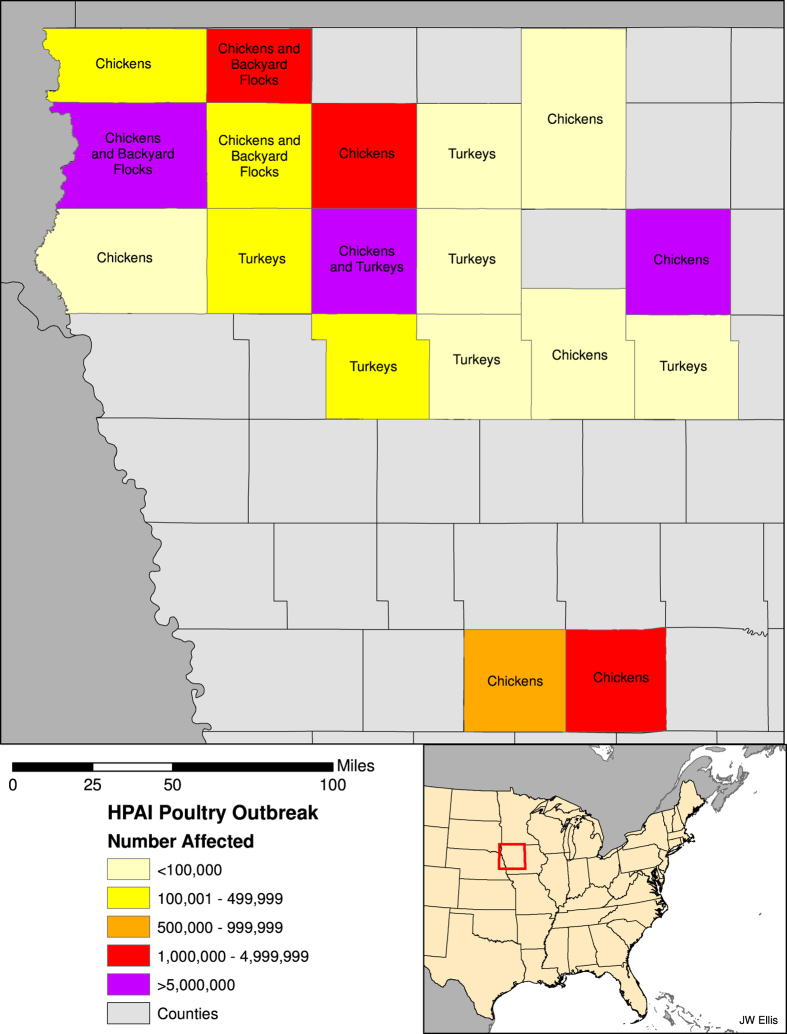
Iowa counties and number of poultry affected by the H5N2 HPAI outbreak in commercial chicken egg-layer facilities in 2015.

**Table 1:** H5N2 HPAI infected premises, flock sizes, outbreak dates, and wildlife sampling dates.

Site	Approximate Flock Size	Date of Clinical Signs	Date H5N2 Confirmed by NVSL	Wildlife Sampling Period
Farm 1	574K	4/28/15	5/11/15	5/13-15/2015
Farm 2	4.1M	4/16/28	4/20/15	5/15-19/2015
Farm 3	275K	5/6/15	5/7/15	5/20-21/2015
Farm 4	275K	4/22/15	4/29/15	5/21-23/2015
Farm 5	3.7M	4/24/15	4/28/15	5/23-27/2015

**Table 2:** Samples collected from wild birds and mammals associated with poultry facilities.

Sample Type	Bird Samples from Infected Sites	Bird Samples from Uninfected Sites	Mammal Samples from Infected Sites	Mammal Samples from Uninfected Sites	Total
Serum	153	99	153	38	443
Oral Swab	217	199	188	38	642
Cloacal Swab	204	196	—	—	400
Nasal Swab/Wash	—	—	188	39	227
External Swab	135	197	26	38	396
Tissue	118	155	207	39	519

**Table 3:** Number of each mammal or bird species captured and sampled at poultry facilities.

Mammal Species	Scientific Name	Infected Farms	Uninfected Farms	Total
House mouse	*Mus musculus*	185	10	195
Deer mouse	*Peromycus maniculatus*	3	19	22
Eastern cottontail	*Sylvilagus floridanus*	2	3	5
Northern short-tailed shrew	*Blarina brevicauda*	0	4	4
Raccoon	*Procyon lotor*	0	3	3
**Bird Species**	**Scientific Name**	**Infected Farms**	**Uninfected Farms**	**Total**
House sparrow	*Passer domesticus*	112	68	180
European starling	*Sturnus vulgaris*	15	54	69
Rock pigeon	*Columba livia*	19	19	38
American robin	*Turdus migratorius*	21	8	29
Common grackle	*Quiscalus quiscula*	12	6	18
Cliff swallow	*Petrochelidon pyrrhonota*	13	1	14
Barn swallow	*Hirundo rustica*	5	11	16

**Table 4:** Summary of avian samples tested for IAV by RRT-PCR and enzyme-linked immunosorbent assay (ELISA) and hemaggluttinin inhibition (HI) tests.

Sample	Species	Site	RRT-PCR	ELISA	HI (icA-H5)
872	American robin	Farm 5	Negative	Suspect positive	≥1:32
881	American robin	Farm 5	Negative	Positive	≥1:32
875	Common grackle	Farm 5	Negative	Positive	<1:8
878	Common grackle	Farm 5	Negative	Positive	<1:8
864	European starling	Farm 5	Negative	Suspect positive	<1:8
870	European starling	Farm 5	Negative	Positive	<1:8*
891	European starling	Farm 5	Negative	Positive	<1:8
892	European starling	Farm 5	RNA detected	Positive	1:16
51	European starling	Uninfected farm	Negative	Suspect positive	<1:8
544	European starling	Uninfected farm	Negative	Suspect positive	<1:8
179	House sparrow	Farm 2	Negative	Suspect positive	<1:8
856	House sparrow	Farm 5	Negative	Positive	<1:8*
585	Rock pigeon	Opportunistic site	Negative	Suspect positive	<1:8
594	Rock pigeon	Opportunistic site	Negative	Suspect positive	<1:8
595	Rock pigeon	Opportunistic site	Negative	Suspect positive	<1:8

Note: HI values ≥ 1:8 are indicative of exposure to an icA-H5 virus.

^*^These samples were negative for icA-H5 but showed limited reactivity to one or both of H7N2 and H9N2, suggesting potential exposure to a N2 virus.
